# Clonal hematopoiesis and hematological malignancy

**DOI:** 10.1172/JCI180065

**Published:** 2024-10-01

**Authors:** William G. Dunn, Matthew A. McLoughlin, George S. Vassiliou

**Affiliations:** 1 Wellcome-MRC Cambridge Stem Cell Institute, University of Cambridge, Cambridge, United Kingdom.; 2 Department of Haematology, Addenbrooke’s Hospital, Cambridge University Hospitals NHS Trust, Cambridge, United Kingdom.

## Abstract

Clonal hematopoiesis (CH), the expansion of hematopoietic stem cells and their progeny driven by somatic mutations in leukemia-associated genes, is a common phenomenon that rises in prevalence with advancing age to affect most people older than 70 years. CH remains subclinical in most carriers, but, in a minority, it progresses to a myeloid neoplasm, such as acute myeloid leukemia, myelodysplastic syndrome, or myeloproliferative neoplasm. Over the last decade, advances in our understanding of CH, its molecular landscape, and the risks associated with different driver gene mutations have culminated in recent developments that allow for a more precise estimation of myeloid neoplasia risk in CH carriers. In turn, this is leading to the development of translational and clinical programs to intercept and prevent CH from developing into myeloid neoplasia. Here, we give an overview of the spectrum of CH driver mutations, what is known about their pathophysiology, and how this informs the risk of incident myeloid malignancy.

## Introduction

Throughout the human lifespan, blood cells are formed continuously through the division and differentiation of cells in a process termed hematopoiesis. At the apex of this hierarchy are a rare population of 50,000–200,000 multipotent hematopoietic stem cells (HSCs) (1), which give rise to more committed progenitors, the progeny of which ultimately generate all blood cell lineages. Somatic mutations are acquired throughout life by all cells, including HSCs. While most are inconsequential or sometimes detrimental, specific rare mutations can confer a fitness advantage that enables the host HSC and its mutation-bearing descendants to expand and occupy a large fraction of the HSC population, a process known as clonal hematopoiesis (CH).

The observation that some elderly healthy female individuals exhibit skewed X-chromosome inactivation patterns provided early evidence of CH (2). In the postgenomics era, several key studies demonstrated that CH is characterized by the presence of recurrent fitness-conferring “driver” mutations in a small number of genes, most of which are associated with leukemia, in individuals without any apparent blood neoplasm (3–6). This process was found to be increasingly prevalent with advancing age, affecting 10%–20% of those aged over 70 years (3, 4, 6), with a subsequent study utilizing next-generation sequencing (NGS) approaches able to detect very small clones identifying CH ubiquitously in people aged 50–60 years (7). A defining feature of CH is its association with an increased risk of incident hematological malignancy, and the magnitude of this risk varies according to the nature of the driver mutation(s), the clonal size, and the context in which CH arises (3, 4, 8–10). This Review will consider what features define CH and how the molecular heterogeneity of CH informs the risk and type of malignant progression.

## The nomenclature of CH

As our understanding of the causes and consequences of CH has grown, it has become clear that the phenomenon exists in several related but distinct forms or subtypes. The terms “clonal hematopoiesis” and “age-related clonal hematopoiesis” (ARCH) refer to any clonal expansion of HSCs, irrespective of the size of the clone. By contrast, “clonal hematopoiesis of indeterminate potential” (CHIP) describes the presence of a CH clone that has expanded to a variant allele fraction of ≥2% (i.e., involving ≥4% of nucleated blood cells), an arbitrary threshold chosen to signify clones large enough to be of potential clinical significance (11). The terms CH, ARCH, and CHIP usually refer to clones associated with somatic driver mutations in genes recurrently mutated in myeloid cancers, while lymphoid CHIP (L-CHIP) describes clonal expansions associated with mutations in lymphoid cancer genes (12). L-CHIP is less common than myeloid CHIP and may originate in committed lymphoid progenitors rather than HSCs. Myeloid and lymphoid clonal expansions do not only arise in association with somatic mutations in individual genes (the focus of this Review) but can also be driven by acquired chromosomal abnormalities, such as amplifications, deletions, and copy neutral loss-of-heterozygosity events. Depending on their specific nature, these mosaic chromosomal aberrations (mCAs) can be associated with an increased risk of myeloid malignancies, lymphoid malignancies, or both (12–18).

By definition, CHIP does not significantly affect blood cell counts but can progress to do so, with the terms clonal cytopenia of undetermined significance (CCUS) (11, 19) and clonal monocytosis of undetermined significance (CMUS) (20) used to describe cytopenia(s) or monocytosis, respectively, in the presence of a CH mutation, in individuals without overt neoplasia, such as a myelodysplastic syndrome (MDS) or chronic myelomonocytic leukemia (CMML).

While a key feature of CH is the presence of a somatic driver mutation, it is recognized that many individuals harbor clonal expansions in the absence of a known driver (“driverless” CH) (3, 21, 22). Studies utilizing somatic “passenger” mutations as barcodes to identify these driverless expansions have shown that driverless CH can be as prevalent as its driver-bearing counterpart but confers a lower risk of malignant progression (3, 23). The nomenclature of CH is summarized in Table 1.

This Review will focus on driver mutation-bearing CH (henceforth referred to as “CH”), examining how the molecular diversity of this entity and the context in which it arises influence the risk of progression to myeloid neoplasia.

## The spectrum of driver mutations in CH

**Figure 1:**
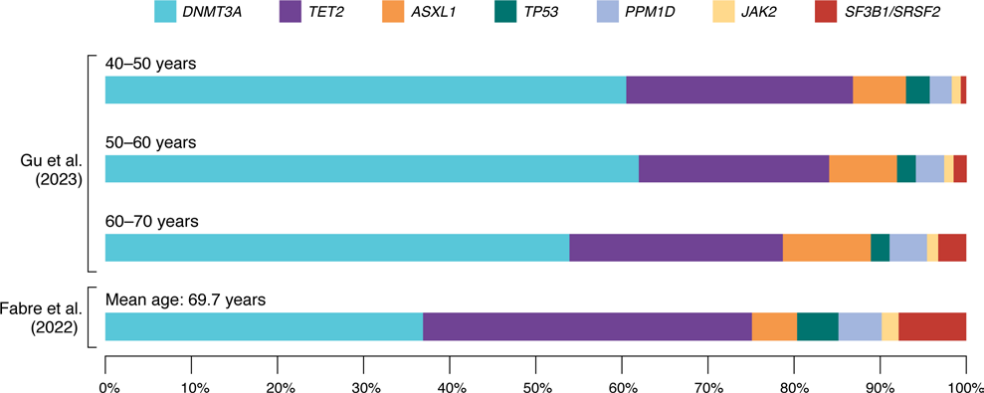
Proportions of commonly mutated CH driver genes in different age ranges. Proportions are based on published data from Gu et al., 2023 (144), broken down by age range, and Fabre et al., 2022 (80). The proportion of individuals with mutations in splicing factor genes (*SF3B1*/*SRSF2*) is very low before the age of 50 but increases markedly with advancing age. Similarly, the relative proportion of DNMT3A-CH decreases with advancing age, as the prevalence of TET2-CH increases. Of note, Fabre et al. use greater sequencing depth, enabling them to identify smaller clones than those in the Gu et al. study.

The majority of CH is caused by mutations in a limited set of genes (Figure 1), predominately those involved in epigenetic regulation (*DNMT3A*, *TET2,* and *ASXL1*), signal transduction (*JAK2*), splicing (*SF3B1*, *SRSF2,* and *U2AF1*), and the DNA damage response (DDR; *TP53* and *PPM1D*). This section will explore current knowledge regarding the molecular consequences of these mutations and their role in driving CH.

### Epigenetic regulation.

The production of mature blood cells requires tightly regulated changes in gene expression that are coordinated by epigenetic marks such as DNA methylation and histone modifications. Mutations in the epigenetic factors *DNMT3A*, *TET2,* and *ASXL1* are found in approximately 70% of CH cases (3, 4, 10) and drive aberrant programs of HSC self-renewal and differentiation.

*DNMT3A* is the most frequently mutated gene in CH, and it encodes a DNA methyltransferase of the *DNMT* family (*DNMT1*, *DNMT3A,* and *DNMT3B*). DNMT3A is the major de novo methyltransferase (24–26) and can form dimers or tetramers with substantially greater activity than the monomer (27–29). DNMT3A deposits methyl groups at the C5 position of cytosines, most commonly those adjacent to a guanine residue (a “CpG” motif), and is usually associated with reduced gene expression (particularly when spanning gene promoters), silencing of transposable elements, and heterochromatin formation. Noncanonical roles for *DNMT3A* have also been reported, including modulation of RNA splicing by promoting spliceosome recruitment (30).

CH-associated mutations in *DNMT3A* are missense or truncating loss-of-function mutations, which lead to global hypomethylation and increased HSC self-renewal (31–35). Approximately 20% of *DNMT3A*-CH involves substitution mutations affecting a single codon, R882 (31). Although *DNMT3A-*R882 mutations are almost always heterozygous, the mutant protein was proposed to exert a dominant-negative effect by dimerizing with its wild-type counterpart and preventing formation of the more active tetrameric complex (25, 36), although this has been disputed (37). The reduced methyltransferase activity in *DNMT3A*-mutated cells causes widespread hypomethylation, including formation of large hypomethylated regions known as methylation “canyons” (38). This prevents the silencing of multipotency genes during hematopoietic differentiation and drives persistent activation of HSC self-renewal programs (25, 33, 35, 39–41). In line with their enhanced self-renewal, *Dnmt3a*-mutated cells outcompete wild-type cells in competitive hematopoietic progenitor transplant experiments (33, 35, 41). Moreover, they retain their ability to produce multilineage output and are immunophenotypically indistinguishable from unmutated cells, although their contribution to mature progeny is lost following successive transplants (41). *DNMT3A* mutations have also been associated with hypermethylation at specific loci (24); however, the relevance of this to clonal expansion is poorly understood.

*TET2* is the second most frequently mutated gene in CH. Belonging to the TET family of α-ketoglutarate (α-KG, also known as 2-oxoglutarate or 2-OG) dependent dioxygenases, TET2 promotes DNA demethylation by converting 5-methylcytosine (5mC) to 5-hydroxymethylcytosine (5hmC) (42, 43). 5hmc is subject to active demethylation via a series of intermediates (42, 44) and passive demethylation due to reduced binding of the maintenance methyltransferase DNMT1 (45, 46). TET2 can also repress inflammatory signaling via HDAC2-associated histone deacetylation (47).

The loss-of-function mutations in *TET2* seen in CH have been studied extensively using mouse models. Similar to *Dnmt3a* mutations, *Tet2*^–/–^ cells exhibit a clonal advantage in competitive transplantation, increased replating capacity in vitro, and a granulocyte-monocyte lineage bias resembling CMML (43, 48–52). These features have been attributed to DNA hypermethylation and subsequent changes in transcription factor binding at loci involved in HSC self-renewal and lineage-specific differentiation (53–55). *Tet2*^–/–^ mice also possess elevated IL-6 levels and reduced apoptosis in response to inflammatory cytokines (47, 56, 57), suggesting an additional feedback loop through which *TET2* mutations may drive CH.

Reduced *TET2* function is also implicated in *IDH1-* and *IDH2-*mutant CH. Located in the cytoplasm (IDH1) or mitochondrial matrix (IDH2), IDH1/2 are metabolic enzymes that convert isocitrate to α-KG. Hotspot mutations in either gene (*IDH1-*R132, IDH2-R140, and *IDH2-*R172) alter enzymatic function and promote the reduction of α-KG to the “oncometabolite” 2-hydroxyglutarate (2-HG) (58–60). The structural similarity between α-KG and 2-HG allows the latter to occupy substrate-binding sites on TET2 and other α-KG–dependent dioxygenases, including prolyl hydroxylases and histone demethylases, inhibiting their enzymatic function (61, 62). Mutations in *IDH1*/*2* and *TET2* are mutually exclusive in hematological malignancy (60), supporting that inhibition of *TET2* activity plays a dominant role in *IDH1/2*-mutant myeloid malignancy. However, in contrast to *TET2*, *IDH1/2* mutations are rare in CH, which may reflect the fact that they are limited to hot spots and also in part that they may progress to myeloid neoplasms (MNs) more rapidly.

*ASXL1* encodes a chromatin-binding protein that interacts with several histone-modifying complexes (63–66), including PRC2, which deposits H3K27me, and BAP1, a deubiquitinase that removes the repressive H2AK119Ub mark. Mutations in *ASXL1* drive approximately 10% of CH (3, 4, 10) and take the form of nonsense or frameshift mutations in the final or penultimate exon, resulting in a truncated protein that lacks the C-terminal histone-binding domain. Whereas knockout of *Asxl1* in mice drives an MDS-like phenotype characterized by increased H3K27me and derepression of *Hoxa* genes (66, 67), *ASXL1* truncating mutations are thought to be gain of function (68) and stabilize interactions between ASXL1 and BAP1 (69–71). This promotes BAP1 deubiquitinase activity, causing a reduction in H2AK119Ub levels and upregulation of a myeloid gene expression program (72). Mouse models of *Asxl1* mutations exhibit several overlapping phenotypes, including myeloid skewing, anemia, and thrombocytosis, and varying rates of progression to overt leukemia. However, the competitive advantage for *Asxl1*-mutated HSCs is more limited compared with *Dnmt3a*- or *Tet2*-mutant cells (73–79).

Mutations in *DNMT3A*, *TET2,* and *ASXL1* have widespread and divergent effects on gene expression yet are all capable of driving clonal expansion. This is particularly surprising with respect to *DNMT3A* and *TET2*, whereby mutations with opposing effects on DNA methylation promote similar increases in HSC fitness and/or “stemness.” Additionally, there are key differences between these CH subtypes, with *DNMT3A* dominating CH in early life and *TET2*-CH becomes increasingly common later in life, overtaking *DNMT3A*-CH by the eighth decade (80, 81). It is also notable that, while smoking increases the risk of most forms of CH, it is most strongly associated with *ASXL1*-CH (10, 82). Finally, lineage contribution studies have shown that *DNMT3A*-CH can more commonly contribute to nonmyeloid lineages compared with other CH subtypes (83).

### Signal transduction.

HSC behavior also depends on the integration of external stimuli, mediated by the binding of cytokines to extracellular receptors and subsequent activation of intracellular signaling. Mutations in components of the erythropoietin (EPO) and thrombopoietin (TPO) signaling pathways, specifically activating mutations in *JAK2*, *CALR,* or *MPL*, can drive aberrant HSC proliferation and clonal expansion and can eventually lead to the development of myeloproliferative neoplasms (MPNs) or MDS/MPN overlap syndromes.

*JAK2* encodes a cytoplasmic kinase that associates with receptors for several cytokines, including EPO, TPO, and G-CSF. Following cytokine binding, JAK2 activates pathways, including JAK/STAT, MAPK, and PI3K/AKT. *JAK2-*V617F hotspot mutations disrupt the pseudokinase domain, resulting in constitutively active JAK2 and downstream activation of JAK/STAT signaling in the absence of cytokine engagement (84–88). This promotes myeloid cell expansion that can lead to the development of MPNs, such as polycythemia vera (PV) or essential thrombocythemia (ET), characterized by the overproduction of red blood cells and/or platelets or primary myelofibrosis (PMF) linked to the proliferation of abnormal profibrotic megakaryocytes (89).

Substitution mutations in *MPL*, encoding the TPO receptor, promote conformational changes resembling those induced by TPO binding and subsequently activate JAK2 (90, 91). Aberrant MPL activation is also seen with frameshift mutations in the final exon of *CALR*, an ER-associated chaperone protein whose mutant form binds MPL and activates JAK/STAT signaling in a MPL-dependent manner (92–99). Whereas *JAK2* mutations mimic signaling from several cytokine receptors and lead to diverse phenotypes, *MPL* and *CALR* mutations are thought to act solely through TPO and are almost exclusively associated with ET and PMF (90, 100, 101). Unlike *JAK2*, *CALR/MPL* mutations are rarely detected in CH, possibly because they promote thrombocytosis, even at low clonal fractions, increasing the likelihood of meeting the diagnostic threshold for ET.

There is a well-established trade-off between proliferation and long-term self-renewal in HSCs, raising questions about how myeloproliferative mutations in *JAK2*, *CALR,* and *MPL* can simultaneously drive HSC expansion over decades. This antagonism is captured in mouse HSCs expressing human *JAK2*-V617F, which exhibit enhanced colony formation at the expense of impaired long-term reconstitution capacity (102, 103). The absence of a long-term competitive advantage over wild-type cells is also seen in *Calr*-mutant mice, despite their development of ET-like disease (97–99). A possible explanation for this is that mutations in *JAK2*, *CALR,* and *MPL* may promote clonal expansion in specific genetic backgrounds or environmental contexts not captured in current models.

### Splicing factors.

The splicing factors *SF3B1*, *SRSF2,* and *U2AF1* are another class of genes frequently mutated in CH, particularly in elderly individuals (6). Splicing describes the removal of intronic sequences from nascent pre-mRNAs to generate a mature mRNA transcript. This process is carried out by the spliceosome, a series of multisubunit ribonucleoprotein complexes. *SF3B1*, *SRSF2,* and *U2AF1* encode core factors within the spliceosome, with SRSF2 involved in exon recognition by binding to exonic splicing enhancers, SF3B1 in branch point recognition and U2AF1 in the recognition of the 3′ splice site (104, 105). Splicing factor gene mutations typically result in single amino acid substitutions at hot spots such as *SRSF2-*P95, *U2AF1-*S34, and *U2AF1-*Q157 and several positions within *SF3B1*, including *SF3B1-*K700, *SF3B1-*K666, and *SF3B1*-R625 (105, 106). These mutations alter protein function, leading to aberrant splicing of large numbers of transcripts and the generation of novel mRNA isoforms. Additional mutations have also been detected in the splicing factor *ZRSR2*, albeit more rarely and as nonsense, frameshift, or substitution mutations that disrupt the function of the minor spliceosome and cause aberrant retention of U12-type introns (106, 107).

Initial investigations of splicing factor mutations deployed knockin mouse models, which develop an MDS-like disease (108–112) but lack some of the characteristic features seen in patients, such as the presence of ring sideroblasts in *SF3B1*-mutated MDS (113, 114). This is in large part due to the limited overlap in mis-spliced transcripts between mice and humans (111), such as the *ABCB7* and *TMEM14C* mRNAs that play important roles in the generation of ring sideroblasts in humans (115, 116). Nevertheless, the presence of an MDS-like phenotype in mice suggests that dysregulated splicing per se may play a role in pathogenesis, regardless of the specific transcripts affected. Recent studies have also shown that splicing factor mutations lead to the generation of R-loops, DNA:RNA hybrids that can activate DDR signaling (117–119). Notably, current models of splicing factor mutation exhibit a reduction in cellular fitness, as assessed by competitive transplantation (108–112), so the mechanism through which splicing factor gene mutations drive clonal expansion remains elusive.

The absence of clonal expansion in models of *SF3B1*, *SRSF2,* and *U2AF1* mutations may relate to the unique relationship these mutations exhibit with age. Unlike other CH drivers, splicing factor gene mutations are rarely detected prior to middle age and show a dramatic increase in prevalence in elderly individuals (6). Going forward, understanding interactions between the pleiotropic effects of splicing factor mutations and an aging hematopoietic system will be critical in characterizing their role in CH.

### DNA damage response.

HSCs are exposed to several forms of DNA damage that can lead to oncogenic mutations and cancer development (120). To safeguard against this, the DDR enables cells to sense genomic insults and activate protective mechanisms. These include cell cycle arrest, giving time for DNA repair, and the activation of apoptosis or senescence if DDR signaling persists. Given the antiproliferative and tumor-suppressive role of the DDR, mutations in this pathway (*TP53*, *PPM1D*) are recognized drivers of CH.

*TP53* encodes a transcription factor, p53, that is typically maintained at low levels in cells via proteasomal degradation (121, 122). Cellular stress such as DNA damage causes phosphorylation and stabilization of p53 (123), which then activates transcriptional programs associated with cell cycle arrest, apoptosis, and senescence (124). In CH, *TP53* mutations are predominantly truncating or missense mutations affecting the DNA-binding domain of the protein (125). These p53 variants are less able to bind DNA or activate transcription, and missense mutations can exhibit a dominant-negative effect by oligomerizing with wild-type p53 and partially reducing its activity (125). As a result, *TP53*-mutant cells exhibit a reduction in apoptosis when exposed to radiation or cytotoxic chemotherapy (120, 121). The antiapoptotic effects of *TP53* mutations explain the increased prevalence of *TP53*-mutant CH after cancer chemo-/radiotherapy and its association with therapy-related MNs (tMNs) (126–129).

Similar prevalence patterns are seen with mutations in *PPM1D* (130), another DNA damage response gene frequently mutated in CH. *PPM1D* expression is induced by p53, and the protein acts in a negative feedback loop, dephosphorylating p53 and other DDR factors and suppressing DDR-mediated apoptosis (131, 132). In CH, frameshift or nonsense mutations distributed within the final exon of *PPM1D* create a C-terminally truncated protein that lacks a ubiquitination domain (9). As a result, truncated PPM1D is several-fold more stable than wild-type PPM1D but maintains its phosphatase activity. The increased levels of the stabilized mutant PPM1D promote dephosphorylation of several DDR factors, suppressing activation of the DDR and reducing apoptosis following cytotoxic chemotherapy or radiation (9, 133). Similar mechanisms likely explain why CH with mutations in other DDR factors (*CHEK2*, *ATM,* and *SRCAP*) are also observed in patients following chemotherapy (127–129, 134).

Notably, a substantial proportion of people with *TP53*- and *PPM1D*-mutant CH lack prior therapy exposure (128). It is possible that the antiapoptotic effects of these mutations promote survival following alternative sources of cellular stress or that these mutations have pleiotropic effects that increase HSC fitness via alternative mechanisms, as suggested for *TP53* (135, 136).

## Associations between CH and hematological malignancy

In 2014, landmark papers from Genovese et al. and Jaiswal et al. established an association between CH and incident hematological malignancies (3, 4). Several studies have since confirmed this association and demonstrated that this is predominantly related to progression to myeloid cancers (8, 10, 137–139). MNs, which encompass acute myeloid leukemia (AML), MDS, and MPNs, represent a family of cancers with a combined annual incidence of approximately 12 cases per 100,000 persons (140). Molecular heterogeneity is a feature of all MN subtypes (141–143), and many of the mutated genes and genomic aberrations found across the spectrum of MN are drivers of CH. As such, CH is regarded as a premalignant lesion, and the relationship between CH and MN can be considered analogous to that between monoclonal gammopathy of undetermined significance (MGUS) and multiple myeloma (MM) or monoclonal B lymphocytosis (MBL) and chronic lymphocytic leukemia (CLL). Similar to MGUS and MBL, CH can precede the development of MN by many years, with an overall annual rate of progression of around 0.5%–1% (4). However, the precise somatic mutations driving each clone strongly influence the likelihood and nature of progression to MN (144).

While *DNMT3A* is the most frequently mutated gene in CH, the risk of transformation of *DNMT3A*-CH is low compared with other CH subtypes. However, this risk is markedly higher for R882 hotspot mutations that are highly fit (145) and 8-fold more likely to progress to AML than non-R882 *DNMT3A*-CH (144). It is also notable that *DNMT3A*-CH was not found to be significantly associated with progression to MDS or MPN (144). Of the other commonly mutated epigenetic modifiers, *TET2*-CH confers a modestly increased risk of incident AML or MPN, while *ASXL1*-CH shows a stronger association with all major subtypes of MN. An important exception is the striking association between *TET2*-CH and incident CMML (hazard ratio, 91.5; based on analyses by Kar, Quiros et al., ref. 10), although the absolute risk of developing CMML from *TET2*-CH is low.

In contrast to CH driven by mutations in epigenetic modifiers, CH driven by mutations in splicing factor genes *SF3B1*, *SRSF2*, and *U2AF1* engenders a much higher risk of progression to MN, particularly MDS (144). Analogously, driver mutations in signal transduction genes *JAK2, CALR,* and *MPL* confer a high risk of progression to MPNs (144). These predilections are unsurprising, since these mutations are defining features of the associated malignancies: for example, *SF3B1* mutations are characteristic of the MDS subtype refractory anemia with ring sideroblasts (RARS), whereas mutations occurring in *JAK2*, *CALR,* and *MPL* are MPN-defining mutations (84, 86, 87, 90, 91, 100, 101, 113, 146, 147).

*TP53*-mutant CH exhibits a variable association with MN in the literature, with some studies demonstrating a high risk of progression (8, 137) and others finding no significant association (144, 148). This heterogenous association appears counterintuitive given that *TP53* mutations confer a dismal prognosis in MDS and AML, and it is likely to be a product of the multitude of confounding factors modulating the behavior and fitness of *TP53*-mutated clones, such as previous exposure to cytotoxic chemotherapy (128), presence of germline pathogenic variants (Li-Fraumeni syndrome) (149), biallelic *TP53* loss (150, 151), and mutation type (with missense variants behaving in a dominant-negative manner) (125). Similarly, gain-of-function mutations in *PPM1D* are present in approximately 20% of cases of therapy-related AML and MDS but are rare in primary (de novo) AML, consistent with prior chemotherapy exposure strongly augmenting the fitness advantage of *PPM1D*-mutant CH clones (9). Where *PPM1D* mutations are present in tMNs, they are often subclonal, suggesting *PPM1D* mutations in this context may indirectly reflect genotoxic damage, rather than being true drivers of the tMN (152).

Beyond the individual mutated gene(s), the number of driver mutations and CH clone size also correlate strongly with progression (8, 137, 144, 148). Furthermore, the propensity to transform is proportional to the relative growth rate associated with individual driver genes: for example, splicing factor mutations, which are among the fittest CH drivers, are associated with a high risk of progression to MN, whereas non-R882 *DNMT3A* mutations typically have a lower fitness effect and progress infrequently (80, 145). An additional factor is the mode of progression from CH to MN. Although CH is the shared precursor of most cases of MN, the trajectory linking asymptomatic clonal expansion (i.e., CH) to frank malignancy varies between different types of MN. De novo AML is usually explosive in its clinical presentation, with previously healthy patients often presenting with complications of fulminant bone marrow failure or leukostasis. Two of the most common drivers of de novo AML are small duplications/insertions in the final exon of *NPM1* and internal tandem duplications of *FLT3* (*FLT3*-ITD). Although these mutations are highly recurrent in AML, they are conspicuously absent in CH. This suggests that acquisition of these mutations leads to rapid clonal expansion and transformation to AML in a short time frame, a trajectory exemplified in a study by Quiros et al., who employed sensitive variant calling to identify individuals with *NPM1* mutations in the United Kingdom Biobank (UKB) (153). Their analysis of 200,453 individuals with whole-exome sequencing (WES) data identified only two with *NPM1* mutations, both of whom developed AML within five months. It is also notable that both these individuals had large antecedent *DNMT3A*-CH clones and that it is common for *DNMT3A* (particularly R882)*, NPM1,* and *FLT3* mutations to co-occur in AML (142, 154); indeed, *NPM1* mutations in isolation are known to be insufficient for transformation to AML (155). Taken together, this implies that CH mutations such as *DNMT3A*-R882 may either facilitate the driver effects of *NPM1* mutations or increase the likelihood of acquiring these and potentially also of *FLT3*-ITD mutations, ostensibly through a shared mutagenic process, since both are insertion mutations with similar molecular anatomies (156, 157).

**Figure 2:**
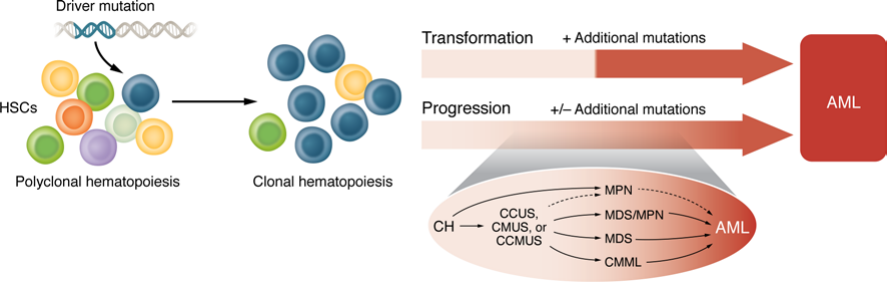
Malignant progression or transformation of CH. The transition between CH and myeloid neoplasia can occur via different routes, including direct “transformation” to de novo acute myeloid leukemia (AML) or “progression” through clonal myeloid disorders, such as clonal cytopenia of undetermined significance (CCUS), clonal monocytosis of undetermined significance (CMUS), clonal cytopenia and monocytosis of undetermined significance (CCMUS), and established myeloid neoplasms, such as the myelodysplastic syndrome (MDS), myeloproliferative neoplasms (MPN), and chronic myelomonocytic leukemia (CMML), or other MDS/MPN overlap syndromes, each of which has potential to progress to (secondary) AML. Dotted lines in the progression inset indicate a less frequent progression path: for example, CCUS predominantly progresses to MDS, but in a minority of cases progresses to MPN (specifically, primary myelofibrosis), while MPNs progress to AML only infrequently. Transformation from CH to AML requires additional leukemia-associated mutations, such as NPM1/FLT3-ITD mutations, whereas acquisition of additional driver mutations is not always observed at progression from CH to MDS or MPN.

In comparison to de novo AML, the distinction between CH and MDS or MPN is less dichotomous (Figure 2). In fact, a proportion of individuals with CH driven by mutations in splicing or signal transduction pathways who progress to MDS or MPN appear to do so without acquiring a further driver event: for example, approximately one-quarter of *SF3B1*-mutated MDS cases display no additional driver mutations (141), and 45% of MPNs bear only a single driver, namely mutant *JAK2*, *CALR,* or *MPL* (143). In this context, the transition between CH and MDS or MPN can be indistinct and may reflect either the clone reaching a critical mass at which it appreciably perturbs blood counts, leading to clinical presentation, or the accumulation of heritable cellular changes that progressively perturb hematopoiesis.

Because of its more gradual nature, the transition from CH to MDS can frequently be identified at the intermediate state of CCUS, where the morphological and cytopenic changes do not meet defined criteria for MDS diagnosis. Similarly, the intermediate phase between CH and CMML has recently been enshrined by the International Consensus Classification as CMUS (see Table 1) (20). By contrast, the diagnostic criteria of PV and ET do not include an equivalent “clonal cytosis” entity. Instead, a small rise in hemoglobin concentration or platelet count that leads to crossing predefined thresholds results in an arbitrary “jump” from CH to MPN (20, 146). This highlights how prevailing pathological classifications do not adequately capture the biological continuum and progression from CH to MDS or MPN.

## Risk stratification of CH and prediction of MN

Despite recent therapeutic advances, survival remains poor for patients with AML and MDS. The observation that myeloid malignancies are preceded by a long, detectable preclinical phase of clonal expansion raises the prospect of intervening within this window to prevent or delay malignant transformation. The current lack of premalignant intervention(s) notwithstanding, a key obstacle to realizing the goal of MN prevention is the high prevalence but low overall progression rate of CH: consequently, when CH is detected, accurate prediction of MN risk is of paramount importance in understanding which patients could benefit from clinical follow-up and/or entry into interventional studies.

Two groups independently sought to address this challenge utilizing the wealth of data available in the UKB. Weeks et al. (148) identified 11,337 cases of CHIP and CCUS among 193,743 UKB participants, labeled each according to whether they developed incident MN within ten years of follow-up, and then used demographic, molecular, and blood count data as features in a decision tree–based machine learning model. Having generated binarized features from their final model, they used these as input to a Cox regression model to generate coefficients from which they derived their CH risk score (CHRS), which designated individuals as being at low, intermediate, or high risk of MN. Through validation in an unseen internal and two external cohorts, they showed that this simple, interpretable score was strongly predictive of incident MN, with individuals with low CHRS having an event rate of 0.6% versus 15.2% for those with high CHRS in one of the external CH cohorts.

In comparison, Gu et al. (144) identified participants with CH and performed forward stepwise Cox regression to fit a parsimonious model (termed MN-predict) on a training cohort of 207,035 individuals to predict MN-free survival using demographic, molecular, blood count, and biochemical data and similarly validated their model in an unseen internal test set and two external CCUS cohorts. In contrast with the approach of Weeks et al., Gu et al. modeled each MN subtype separately; made no distinction among CH, CHIP, or CCUS; and generated quantitative, time-dependent predictions of MN-free survival for each MN subtype (AML, MDS, MPN).

**Figure 3:**
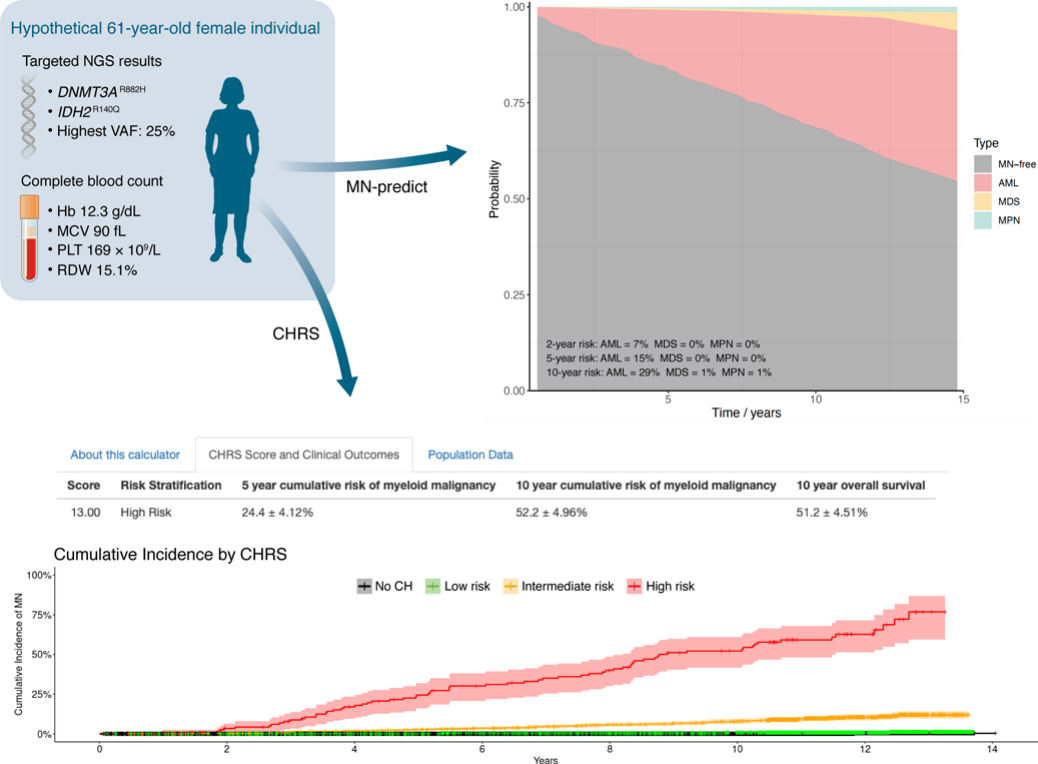
Risk stratification of CH. A hypothetical individual with *DNMT3A* and *IDH2*-mutated CH is used as an exemplar. NGS, demographic, and complete blood count data are used as inputs into two CH risk stratification tools (144,148), and both identify the individual as being at high risk of incident MN. The clonal hematopoiesis risk score (CHRS) (148) (available online at http://www.chrsapp.com/) designates the individual as “high risk” and depicts the cumulative incidence of myeloid neoplasia for this subset. The output of MN-predict (144) (available online at https://bioinf.stemcells.cam.ac.uk/shiny/vassiliou/MN_predict) shows the probability of progression to each MN subtype, highlighting that the risk is predominantly related to transformation to AML in this sample case. Images from CHRS and MN-predict tools were reproduced with permission. Hb, hemoglobin; MCV, mean corpuscular volume; PLT, platelet count; RDW, red cell distribution width.

Each approach has its own merits: the CHRS developed by Weeks et al. offers a readily interpretable score and categorization of the risk of any MN that will be easily understood by clinicians (148), whereas the MN-predict approach adopted by Gu et al. offers nuanced, continuous estimates of the likelihood of developing each type of the three main types of MN (144) (Figure 3). CHRS takes as input a smaller number of variables commonly used in the clinic, whereas MN-predict can also incorporate other variables that may not be routinely measured in clinical practice, although it can still generate robust predictions in their absence. Furthermore, only MN-predict offers predictions for CH driven by mutations in *U2AF1*, since issues with this locus in the human reference genome (158) precluded its inclusion in the CHRS approach. Both approaches are readily accessible through user-friendly web interfaces (http://www.chrsapp.com and https://bioinf.stemcells.cam.ac.uk/shiny/vassiliou/MN_predict/) and will be important tools for risk-stratifying individuals identified as having CH, CHIP, or CCUS in the future (though currently neither tool has been prospectively validated nor reviewed by the United States Food and Drug Administration or an equivalent national body). In the absence of a unified risk-stratification strategy for CH, the use of both models, or the development of a model incorporating the best aspects of both may be beneficial in assessing MN risk in individuals harboring CH.

## Clinical management of CH and preventative medicine

With increasingly widespread use of NGS for diagnostics and research, CH is a frequent incidental finding, leading to the establishment of clinics dedicated to managing CH. As CH is a highly prevalent phenomenon in advanced age, adoption of the aforementioned risk-stratification tools in clinical decision-making will be key to ensuring that individuals with high-risk CH are prioritized for closer follow-up and consideration for recruitment to interventional studies. Importantly, knowledge of the clinical context, which cannot be an input feature in predictive models, will be critical in guiding nuanced management of individuals whose age, fitness, perception of risk, comorbidities, treatment history, and other factors will need to be considered prior to deciding their clinical management.

**Figure 4:**
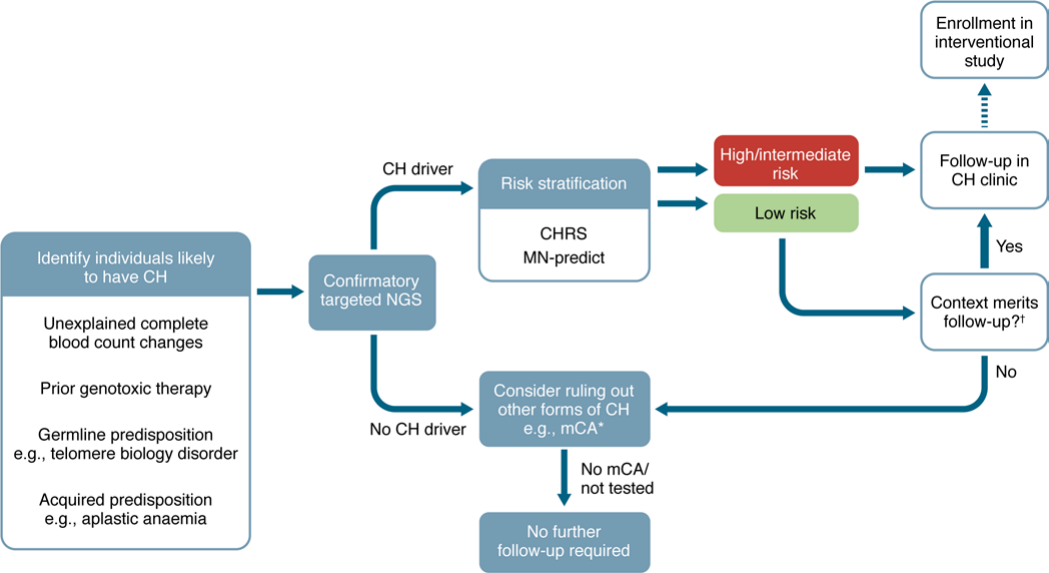
Identification and management of CH. In the absence of population-level screening for CH, testing can be targeted to individuals at higher a priori risk of CH. These include individuals with germline predisposition, such as those with telomere biology disorders, and conditions associated with acquired predisposition to CH, such as acquired aplastic anemia (in which clonal selection occurs as a means of escaping immune-mediated destruction). Furthermore, individuals with certain unexplained complete blood count changes may harbor particular forms of CH, while exposure to cytotoxic chemotherapy or radiotherapy favors the expansion of *PPM1D*- and *TP53*-mutated clones. As most individuals with CH will never develop MN, clinical follow-up and consideration for enrollment into interventional studies should be reserved for individuals at higher risk of malignant progression. *In individuals with no evidence of CH on targeted NGS, testing for mosaic chromosomal abnormalities (mCA) may be considered where there remains a strong suspicion of this form of CH, e.g., in an individual with unexplained cytopenias or monocytosis. †In some cases, individuals designated as “low-risk CH” using risk-stratification tools, may merit follow-up owing to the particular clinical context: a typical example would be an individual with a small (low variant allele fraction [VAF]) *TP53*- or *PPM1D*-mutant clone who is scheduled to undergo chemo- or radiotherapy.

At present, there are no approved therapies to prevent progression of CH to MN, and the fact that individuals with CH are largely asymptomatic dictates that any such intervention must be well-tolerated with minimal toxicity. Furthermore, given the protracted window between CH detection and onset of MN, use of MN-free survival may be an impractical endpoint for initial interventional studies, necessitating the use of surrogate endpoints such as reduction in clone size or improvement in blood indices in the case of CCUS. Recently initiated clinical trials are investigating potential treatments for CH/CCUS, including anti–IL-1β antibody canakinumab for high-risk CCUS (to attenuate inflammation-driven clonal expansion) (159) (NCT05641831), the administration of vitamin C (a cofactor of TET2) for *TET2*-CCUS (NCT03418038), and the exploration of targeted therapies such as IDH1/2 inhibitors in *IDH1/2*-CCUS (NCT05030441 and NCT05102370). In the absence of established therapies, an important role of CH clinics is to identify patients for entry into clinical studies of MN prevention. Furthermore, in practice, CH clinics will establish the mode and frequency of monitoring high-risk CH/CCUS to identify patients who have transitioned to an established MN at the earliest possible stage, until evidence for treating specific subtypes of CH/CCUS is firmly established. A proposed structure for the clinical management of CH focused on MN prevention is outlined in Figure 4.

## Unanswered questions and challenges

Despite the huge advances over the last decade, there are many unknowns in the field of driver gene CH and myeloid cancer prevention. A key unsolved problem is how to detect CH at scale. At present CH is identified by NGS, which is both expensive and impractical to apply at scale to unselected populations to identify the few individuals at high risk of MN. In an attempt to address this, efforts have been made to reduce the cost of CH-targeted NGS (160, 161), but applying this at scale in asymptomatic individuals would still be practically challenging. An alternative approach is to target NGS testing to individuals with a higher a priori risk of harboring high-risk CH or CCUS on the basis of simpler, more scalable tests. Since CH is known to perturb blood indices, such as red cell distribution width and mean cell volume, one such approach might be to leverage complete blood count data to identify individuals likely to harbor CH who can be prioritized for genetic testing.

Prediction of progression from CH to de novo AML also remains challenging, despite the advances culminating in the CHRS and MN-predict risk stratification tools. For example, the predictive performance of MN-predict was lower for AML than MDS and MPN, and this likely reflects the varying trajectories of CH progression to different MN subtypes, with transformation to de novo AML being more rapid. Since outcomes for AML remain poor, refining risk stratification tools to specifically improve de novo AML prediction could be a priority in coming years.

Finally, while the landscape of recurrent driver mutations has been established by applying NGS to large cohorts, the precise mechanism by which many of these mutations engender a fitness advantage to mutant HSCs is unclear, and this will be of paramount importance in designing targeted treatments to avert progression of CH to MN. Most strikingly, the fitness effect of splicing factor mutations, which drive rapid clonal expansion and confer high risk of progression while perturbing a critical cellular process, is yet to be established: paradoxically, these clones do not expand well in vitro or in vivo and seem to expand preferentially in advanced age, as hematopoiesis becomes increasingly oligoclonal (6, 21, 110, 111). Ascertaining the mechanisms through which disruption of critical molecular processes (RNA splicing and DNA methylation) allows cells to expand clonally is likely to be a critical step toward developing rational strategies for myeloid cancer prevention.

## Concluding remarks

The past decade has borne witness to unprecedented advances in our understanding of the natural history and evolution of myeloid cancers, aided substantially by the description and comprehensive molecular characterization of CH. As the use of NGS becomes increasingly commonplace in clinical practice, the number of individuals identified with CH will continue to grow. Only a minority of these individuals will go on to develop MN, and advances in risk stratification are enabling clinicians to quantify the magnitude and nature of this risk. Further improvements in our ability to identify those at highest risk of progression will be important, but the most important hurdle in the successful development of the field of myeloid cancer prevention is the lack of an approved therapy to avert or delay MN development. As the second decade of research into CH commences, the focus is turning toward an improved understanding of the basis of clonal expansion and malignant progression, with the ultimate aim of developing effective interventions that can change the natural history of high-risk CH.

## Figures and Tables

**Table 1:**
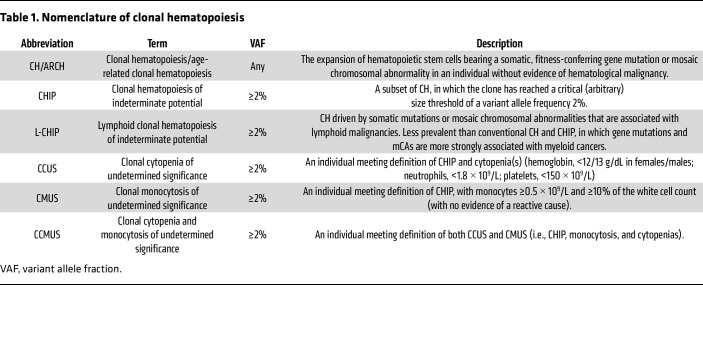
Nomenclature of clonal hematopoiesis

## Version 1 10/01/2024

Electronic publication
